# Prognostic Value of In-Hospital Nutritional Status Improvement in Heart Failure: Insights From JROADHF-NEXT Registry

**DOI:** 10.5334/gh.1534

**Published:** 2026-03-13

**Authors:** Toshitaka Okabe, Keisuke Kida, Nobuyuki Enzan, Masataka Ikeda, Takahiro Okumura, Takeshi Kitai, Takeshi Tohyama, Tatsunori Taniguchi, Shouji Matsushima, Yuya Matsue, Hiroyuki Tsutsui

**Affiliations:** 1 Division of Cardiology, Showa Medical University Northern Yokohama Hospital, Kanagawa, Japan; 2 Department of Pharmacology, St. Marianna University School of Medicine, Kawasaki, Japan; 3 Department of Cardiovascular Medicine, Faculty of Medical Sciences, Kyushu University, Fukuoka, Japan; 4 Division of Cardiovascular Medicine, Research Institute of Angiocardiology, Faculty of Medical Sciences, Kyushu University, Japan; 5 Cardiovascular Disease Initiative, Broad Institute, Cambridge, MA, United States; 6 International University of Health and Welfare, Okawa, Japan; 7 Department of Cardiovascular Medicine, Kyushu University Beppu Hospital, Beppu, Oita, Japan; 8 Department of Cardiology, Nagoya University Graduate School of Medicine, Nagoya, Japan; 9 Department of Advanced Cardiovascular Therapeutics, Nagoya University Graduate School of Medicine, Nagoya, Japan; 10 Department of Cardiovascular Medicine, National Cerebral and Cardiovascular Center, Osaka, Japan; 11 International University of Health and Welfare, Okawa, Japan; 12 Institute for Medical Engineering and Science, Massachusetts Institute of Technology, Cambridge, MA, United States; 13 Department of Cardiovascular Medicine, Osaka University Graduate School of Medicine, Osaka, Japan; 14 Department of Cardiovascular Biology and Medicine, Juntendo University Graduate School of Medicine, Tokyo, Japan

**Keywords:** heart failure, malnutrition, nutritional status, CONUT score, prognosis

## Abstract

**Background:**

Malnutrition is common in heart failure (HF) and is associated with poor outcomes; however, longitudinal changes in the nutritional status of patients with HF are poorly investigated.

**Objectives:**

To assess the prognostic impact of changes in Controlling Nutritional Status (CONUT) score and identify predictors of malnutrition improvement in hospitalized patients with HF.

**Methods:**

We analyzed data on 4,016 patients from a nationwide acute HF registry in Japan (UMIN ID: UMIN000036592). We identified 812 patients with moderate or severe malnutrition at admission (CONUT score ≥5) and stratified them into an improvement (IMP, n = 168) or non-improvement (Non-IMP, n = 644) group based on in-hospital change in CONUT score. The primary outcome was all-cause mortality; the secondary outcome was a composite endpoint of all-cause mortality and HF rehospitalization.

**Results:**

Over a median follow-up of 712 days (IQR, 392–768 days), all-cause mortality was significantly lower in the IMP group than in the Non-IMP group (11.90% vs. 30.12%; log-rank P < 0.0001). The composite endpoint was also lower in the IMP group (29.76% vs. 47.98%; log-rank P < 0.0001). After propensity score matching, the IMP group had consistently lower all-cause mortality and composite endpoints than the Non-IMP group (log-rank P = 0.0002; log-rank P = 0.041). Multivariable Cox proportional hazards model for all-cause mortality with overlap weighting demonstrated that CONUT improvement was associated with lower all-cause mortality (HR, 0.357; 95% CI, 0.205–0.624; P = 0.0003).

**Conclusion:**

In hospitalized patients with acute HF and moderate to severe malnutrition, improvement in CONUT score during hospitalization was associated with lower post-discharge mortality and rehospitalization.

## Graphical Abstract

**Figure:**
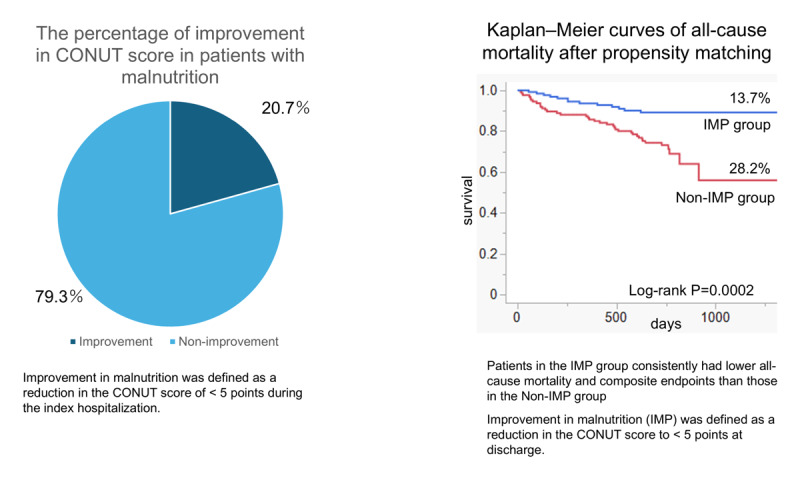


## Introduction

Although guideline-directed medical therapy is strongly recommended to improve survival rate and quality of life in patients with heart failure (HF), recent guidelines also highlight the importance of non-pharmacological management and comprehensive patient assessment, including evaluation of nutritional status (1,2,3). Despite these recommendations, robust evidence supporting the efficacy of nutritional supplementation in patients with HF remains limited (1,2,3).

Malnutrition is frequently observed in patients with HF, affecting 8%–57% of cases, depending on disease severity, and is associated with markedly high mortality (4,5). The heightened susceptibility to malnutrition is attributable to pathophysiological mechanisms associated with HF. Elevated central venous pressure leads to intestinal congestion, resulting in anorexia, diarrhea, and impaired nutrient absorption. Additionally, chronic inflammation in HF promotes a hypercatabolic state, further accelerating nutritional depletion (6). Consequently, careful nutritional assessment has become increasingly important in the management of patients with HF.

The Controlling Nutritional Status (CONUT) score is a widely used and validated tool for assessing the nutritional status of hospitalized patients (7). It can be easily calculated using routine blood test parameters. Several nutritional scoring systems have been shown to predict outcomes of various malignancies (4,5,6,7,8,9). Previous small-scale studies have suggested that the CONUT score is associated with clinical outcomes in patients with HF (6,10). However, robust evidence from large-scale, well-characterized cohorts is still lacking. In particular, the prognostic relevance of longitudinal changes in nutritional indices has not been thoroughly investigated. To address these gaps, we conducted a sub-analysis of the prospective Japanese Registry of Acute Decompensated Heart Failure (JROADHF-NEXT). This study aimed to evaluate whether changes in nutritional status during hospitalization, as assessed by the CONUT score, are associated with post-discharge outcomes (all-cause mortality and HF hospitalization), and to identify factors associated with improvement in patients’ nutritional status.

## Methods

### Study population and design

This retrospective sub-analysis included patients from the JROADHF-NEXT registry (8) with moderate to severe malnutrition (CONUT score ≥5 points at admission) who survived to hospital discharge. JROADHF-NEXT is a prospective multicenter, nationwide registry for acute decompensated HF (UMIN ID: UMIN000036592). This registry included 4,016 patients from 87 institutions in Japan between February 2019 and June 2021. This study was conducted in accordance with the principles of the Declaration of Helsinki. The study protocol using the JROADHF-NEXT database was approved by the institutional review boards of Kyushu University (2019–023), the International University of Health and Welfare (24-KS-005), and all participating hospitals, including Showa Medical University Northern Yokohama Hospital (2025–0068). The study design and primary findings have been previously published (11). Patients with acute decompensated HF, admitted to the participating institutions who were aged ≥20 years, had elevated B-type natriuretic peptide (BNP) ≥100 pg/mL or N-terminal pro-B-type natriuretic peptide (NT-proBNP) ≥300 pg/mL at admission, and provided written informed consent were included. The definition of HF was based on the guidelines for the treatment of acute HF (12). Major exclusion criteria included in-hospital death (since follow-up began at discharge), history of heart transplantation, acute coronary syndrome requiring coronary intervention, and cases deemed inappropriate for registry inclusion by the attending physician at each institution.

Patients with moderate or severe malnutrition were divided into two groups based on changes in their malnutrition status, as assessed using the CONUT score at admission and discharge: the CONUT improvement (IMP) group and the CONUT non-improvement (Non-IMP) group. Patients with missing data for nutritional variables were excluded from the analysis.

### CONUT score assessment

The CONUT score is a screening tool that provides an objective and simple assessment of the nutritional status of hospitalized patients. It is determined from serum albumin, total cholesterol (TC), and peripheral lymphocyte counts (7). Scores were assigned as follows: 1) serum albumin concentration: ≥3.5, 3.0–3.49, 2.5–2.99, <2.5 g/dL were scored as 0, 2, 4, 6, respectively; 2) total lymphocyte count: ≥1.600, 1.200–1.599, 0.800–1.199, <0.800 × 10^9^/L were scored as 0, 1, 2, 3, respectively; 3) TC concentration: ≥180, 140–179, 100–139, <100 mg/dL were scored as 0, 1, 2, 3, respectively. The CONUT score was calculated as the sum of the three component scores. We enrolled patients with a CONUT score ≥5 points at admission, which was defined as at least moderate malnutrition. Improvement in malnutrition was defined as a reduction in the CONUT score to <5 points at discharge.

### Study outcomes

The primary outcome of this study was all-cause mortality within 2 years of discharge, because it was predefined as the primary endpoint of the JROAD-NEXT registry and allows for robust outcome ascertainment. The secondary outcome was a composite endpoint of all-cause mortality or first hospitalization for HF within 2 years after discharge. These outcomes were compared between the IMP and non-IMP groups.

### Statistical analysis

Continuous parameters are presented as mean ± standard deviation (SD) or median (interquartile range, [IQR]), as appropriate. Categorical parameters are presented as counts and percentages. Baseline characteristics were compared between the IMP and the Non-IMP groups using the unpaired t-test or Wilcoxon rank-sum test for continuous variables and the chi-square test or Fisher’s exact test for categorical variables, as appropriate. The Kaplan–Meier method and log-rank test were used to compare survival curves for all-cause mortality and the composite endpoint of all-cause mortality or first hospitalization for HF before and after propensity score matching. Propensity score was estimated using a logistic regression model. Independent variables included in the logistic regression model for calculating propensity score were baseline characteristics that showed statistically significant differences between patients in the IMP and the Non-IMP groups as well as those considered clinically relevant. Specifically, the propensity score model included age, sex, diabetes mellitus, hypertension, estimated glomerular filtration rate (eGFR), hemoglobin, left ventricular ejection fraction (LVEF), tricuspid regurgitation pressure gradient (TRPG), and statin therapy at admission. In most cases, only one BNP or NT-pro BNP level was measured; therefore, these variables were not included in the propensity score model. Propensity score matching was conducted in a 1:1 ratio between the IMP and the Non-IMP groups using nearest-neighbor matching without replacement, with a caliper width set to <0.05 times the standard deviation of the logit of the propensity score (13). To evaluate covariate balance between the groups, standardized mean differences (SMDs) were calculated, with an SMD <0.20 considered indicative of adequate balance.

For sensitivity analysis, a multivariable Cox proportional hazards model with overlap weighting was performed utilizing a simplified set of covariates, including age, sex, IMP status, and a combined log-transformed natriuretic peptide (14,15), which was derived from BNP or NT-proBNP according to data availability. In addition, subgroup analyses were performed to assess the consistency of the association between CONUT improvement and all-cause mortality across clinically relevant subgroups, including sex (male vs. female), age (<75 vs. ≥75 years), anemia (hemoglobin <12 g/dL in women and <13 g/dL in men), chronic kidney disease (eGFR <60 ml/min/1.73 m² vs. ≥60 ml/min/1.73 m²), and natriuretic peptide levels (high vs low, dichotomized at the median of log-transformed values [6.627]). Hazard ratios (HRs) were estimated with Cox proportional hazards models using overlap weighting. Interaction terms were tested with Wald statistics. Detailed results are provided in the Figure S1. Additionally, least absolute shrinkage and selection operator (LASSO) logistic regression analysis with 10-fold cross-validation was performed to identify significant predictors of improvement in malnutrition, as assessed based on the CONUT score during hospitalization. The penalty parameter (λ) was chosen at the minimum cross-validated error (λmin) for the primary analysis, with the 1-SE rule (λ1se) used for sensitivity analysis. LASSO at λmin retained body mass index (BMI) and use of loop diuretic at admission, and the 1-SE rule yielded the same two predictors. These variables (BMI and use of loop diuretics at admission) were subsequently entered into a multivariate logistic regression model with age and sex to assess their independent association with improvement in malnutrition. All statistical analyses were performed using JMP version 17 (SAS Institute Inc., Cary, NC, USA) and R software version 4.5.1 (R Project for Statistical Computing). In the present analysis, complete case analysis was conducted when there was missing data.

## Results

### Patient characteristics

Of the 4,016 registered patients, 365 were excluded because of missing CONUT score at admission or discharge, and 2,839 patients without malnutrition based on the CONUT score on admission were also excluded. Ultimately, 812 patients (IMP group, n = 168; Non-IMP group, n = 644) were included in the final analysis. The patient characteristics are summarized in Table 1. At admission, the CONUT score was 5.863 ± 1.183 in the IMP group and 6.315 ± 1.453 in the Non-IMP group (P = 0.0001), whereas at discharge, it was 3.560 ± 0.498 and 6.943 ± 1.722, respectively (P < 0.0001), resulting in a significantly mean decrease in CONUT score in the IMP group (–2.304 ± 1.289) compared with Non-IMP group (+0.627 ± 1.765; P < 0.0001). Patients in the Non-IMP group were older than those in the IMP group (76.60 ± 12.25 years vs. 69.13 ± 15.63 years, p < 0.0001). Patients in the Non-IMP group had a lower eGFR (41.680 ± 21.41 mL/min/1.73 m^2^ vs. 51.35 ± 24.06 mL/min/1.73 m^2^, P < 0.0001) and hemoglobin levels than those in the IMP group (10.89 ± 2.31 g/dL vs. 11.65 ± 2.23 g/dL, P = 0.0002). Regarding echocardiography parameters, LVEF was higher in the Non-IMP group than in the IMP group (47.06 ± 16.48% vs. 43.44 ± 16.81%, P = 0.013). Although tricuspid annular plane systolic excursion (TAPSE) was similar between the Non-IMP and IMP groups, TRPG tended to be higher in the Non-IMP group than in the IMP group; however, the difference was not statistically significant. BNP levels were comparable between the groups, although NT-proBNP levels differed. Notably, NT-proBNP was measured in 40.14% of cases, while BNP was measured in 69.21% of cases.

**Table 1:** Patient characteristics before propensity score matching.


	NON-IMP	IMP	P VALUE
	
	n = 644	n = 168	

Age, years	76.60 ± 12.25	69.13 ± 15.63	<0.0001

Female, n (%)	225 (34.94)	67 (39.88)	0.237

History of hospitalization of HF, n (%)	276 (42.86)	61 (36.31)	0.123

Hypertension, n (%)	447 (69.41)	103 (61.31)	0.048

Diabetes mellitus, n (%)	279 (43.32)	56 (33.33)	0.018

Dyslipidemia, n (%)	248 (38.51)	57 (33.93)	0.272

Hyperuricemia, n (%)	209 (32.45)	952 (30.95)	0.710

Chronic kidney disease, n (%)	348 (54.04)	67 (39.88)	0.001

Anemia, n (%)	255 (39.60)	45 (26.79)	0.002

Ischemic heart disease, n (%)	257 (39.91)	42 (25.00)	0.0003

Atrial fibrillation, n (%)	297 (46.12)	72 (42.86)	0.449

BMI, kg/m^2^	23.30 ± 4.82	24.10 ± 5.16	0.061

Systolic blood pressure, mm Hg	133.79 ± 29.12	133.71 ± 33.92	0.978

Diastolic blood pressure, mm Hg	77.56 ± 19.30	81.34 ± 20.20	0.025

Heart rate, bpm	88.09 ± 23.54	91.53 ± 21.04	0.086

NYHA3/4	588 (91.31)	153 (91.07)	0.646

Dyspnea, n (%)	580 (90.06)	149 (88.69)	0.605

S3, n (%)	151 (23.45)	48 (28.57)	0.175

Rales, n (%)	281 (43.63)	66 (39.29)	0.309

Eedema, n (%)	482 (74.84)	130 (77.38)	0.494

Anorexia, n (%)	117 (18.17)	40 (23.81)	0.106

medication on admission			

ACEI, n (%)	124 (19.25)	38 (22.62)	0.337

ARB, n (%)	178 (27.64)	36 (21.43)	0.100

Beta blockers, n (%)	334 (51.86)	78 (46.43)	0.210

MRA, n (%)	192 (29.81)	56 (33.33)	0.381

Loop diuretics, n (%)	390 (60.56)	95 (56.55)	0.347

Thiazides, n (%)	30 (4.66)	4 (2.38)	0.162

Tolvaptan, n (%)	145 (22.52)	28 (16.67)	0.092

Nitrates, n (%)	71 (11.02)	5 (2.98)	0.0004

Calcium channel blockers, n (%)	223 (34.63)	51 (30.36)	0.294

Inotropes, n (%)	49 (7.61)	13 (7.74)	0.955

SGLT2I, n (%)	44 (6.83)	10 (5.95)	0.680

DOAC, n (%)	178 (27.64)	42 (25.00)	0.490

Antiplatelet, n (%)	206 (31.99)	33 (19.64)	0.001

ARNI, n (%)	3 (0.83)	0 (0.0)	0.220

Statin, n (%)	241 (37.42)	49 (29.17)	0.044

Laboratory data			

White blood cell, /μL	7030.37 ± 3294.30	7274.74 ± 3332.70	0.393

Lymphocyte,/μL	975.94 ± 686.37	898.33 ± 500.77	0.170

hemoglobin, g/dL	10.89 ± 2.31	11.65 ± 2.23	0.0002

Hematocrit, %	33.42 ± 6.89	35.63 ± 6.52	0.0002

Blood urea nitrogen, mg/dL	30.28 ± 16.71	25.20 ± 14.85	0.0004

Creatinine, mg/dL	1.628 ± 1.304	1.291 ± 0.874	0.0016

eGFR, mL/min/173 m^2^	41.680 ± 21.41	51.35 ± 24.06	<0.0001

Uric acid, mg/dL	6.956 ± 2.428	6.955 ± 2.628	0.996

Sodium, mEq/L	138.62 ± 5.21	138.64 ± 5.54	0.964

Potassium, mEq/L	4.195 ± 0.667	4.071 ± 0.622	0.029

Chloride, mEq/L	103.56 ± 5.465	102.85 ± 5.862	0.144

Total protein, g/dL	6.350 ± 0.776	6.216 ± 0.676	0.041

Albumin, g/dL	3.11 ± 0.46	3.23 ± 0.42	0.0023

Total bilirubin, mg/dL	1.065 ± 0.927	1.274 ± 1,140	0.014

Total cholesterol, mg/dL	136.65 ± 40.73	145.52 ± 31.59	0.001

Triglycerides, mg/dL	79.98 ± 41.59	80.79 ± 40.47	0.823

HDL-cholesterol, mg/dL	45.06 ± 14.64	46.91 ± 14.72	0.155

LDL-cholesterol, mg/dL	75.04 ± 32.51	81.67 ± 27.31	0.018

BNP, pg/mL	1055.51 ± 961.91	1110.36 ± 950.36	0.584

NT-proBNP, pg/mL	11867.01 ± 16417.64	7245.56 ± 6576.99	0.023

CONUT score on admission	6.315 ± 1.453	5.863 ± 1.183	0.0001

CONUT score at discharge	6.943 ± 1.722	3.560 ± 0.498	<0.0001

Echocardiographic parameter			

LV end-diastolic diameter, mm	51.37 ± 9.84	52.49 ± 9.80	0.197

LV systolic diameter, mm	39.00 ± 11.82	41.14 ± 12.30	0.043

LV ejection fraction, %	47.06 ± 16.48	43.44 ± 16.81	0.0134

LA diameter, mm	44.59 ± 8.85	44.65 ± 11.00	0.937

LA volume, mL	91.90 ± 53.58	99.85 ± 69.84	0.203

IVST, mm	9.98 ± 2.35	9.69 ± 2.40	0.169

PWT, mm	9.89 ± 2.05	10.02 ± 2.54	0.480

E wave, cm/s	86.22 ± 33.81	86.43 ± 36.19	0.946

A wave, cm/s	73.67 ± 32.29	64.60 ± 26.93	0.011

TAPSE	16.48 ± 4.83	15.96 ± 4.51	0.353

TRPG, mm Hg	30.18 ± 12.92	27.82 ± 13.06	0.050

medication at discharge			

ACEI, n (%)	226 (35.09)	81 (48.21)	0.002

ARB, n (%)	179 (27.80)	46 (27.38)	0.915

Beta blockers, n (%)	476 (73.91)	134 (79.76)	0.112

MRA, n (%)	353 (54.81)	110 (65.48)	0.0122

Loop diuretics, n (%)	542 (84.16)	143 (85.12)	0.760

Thiazides, n (%)	45 (6.99)	8 (4.76)	0.281

Tolvaptan, n (%)	260 (40.37)	57 (33.93)	0.125

Nitrates, n (%)	47 (7.30)	7 (4.17)	0.127

Calcium channel blockers, n (%)	214 (33.23)	57 (33.93)	0.864

Inotropes, n (%)	66 (10.25)	20 (11.90)	0.540

SGLT2I, n (%)	110 (17.08)	33 (19.64)	0.442

DOAC, n (%)	236 (36.65)	63 (37.50)	0.838

Antiplatelet, n (%)	249 (38.66)	47 (27.98)	0.009

ARNI, n (%)	19 (2.95)	2 (1.19)	0.139

Statin, n (%)	282 (43.79)	57 (33.93)	0.020


Data are presented as mean ± SD or n (%). **Abbreviations:** ACEI, angiotensin-converting enzyme inhibitors; ARB, angiotensin II receptor blockers; ARNI, angiotensin receptor-neprilysin inhibitors; BNP, B-type natriuretic peptide; CONUT, Controlling Nutritional Status; DOAC, direct oral anticoagulants; GFR, glomerular filtration rate; HF, heart failure; IVST, interventricular septal thickness; LA, left atrial; LV, left ventricular; MRA, mineralocorticoid receptor antagonists; NYHA, New York Heart Association; PWT, posterior wall thickness; SGLT2I, sodium-glucose cotransporter 2 inhibitors; TAPSE, tricuspid annular plane systolic excursion; TRPG, tricuspid regurgitation pressure gradient.

### Clinical outcomes

**Figure 1:**
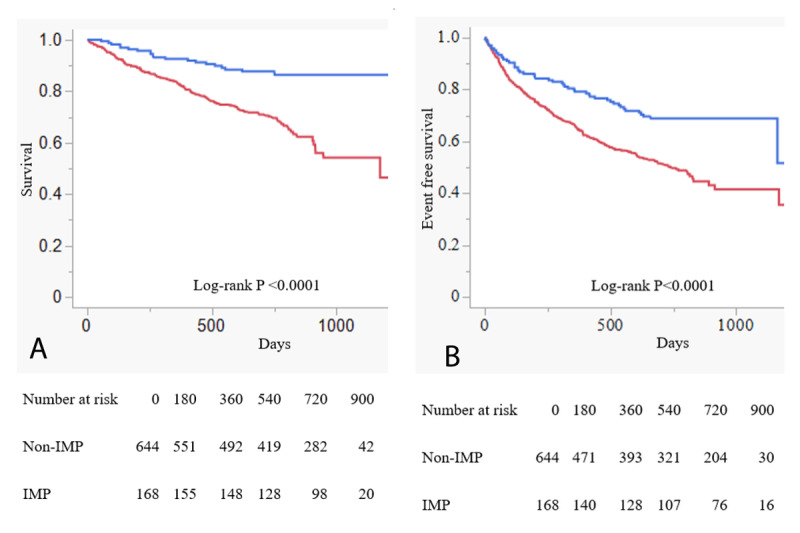
Kaplan–Meier curves of primary and secondary endpoints before propensity matching. **A.** Kaplan–Meier survival curves of all-cause mortality in patients with heart failure according to group assignment. The IMP group is shown in blue and the Non-IMP group in red. **B.** Kaplan–Meier survival curves of composite endpoint (all-cause mortality or hospitalization for heart failure) in patients with heart failure according to group assignment. The IMP group is shown in blue and the Non-IMP group in red.

**Figure 2:**
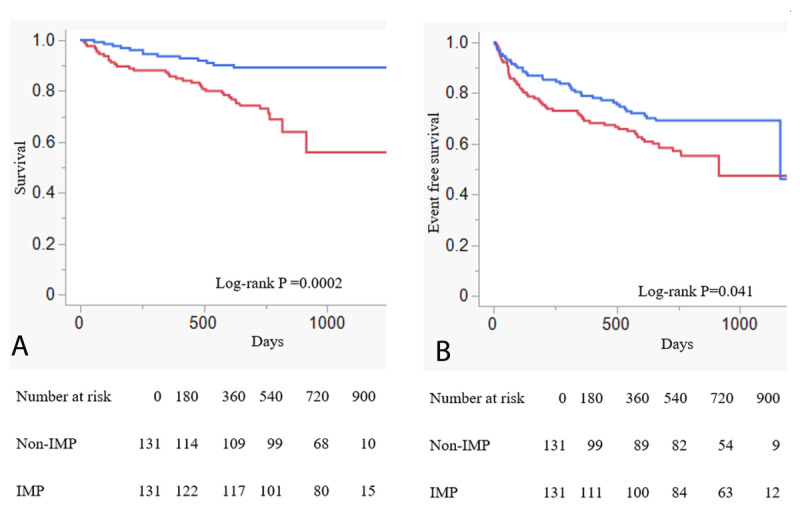
Kaplan–Meier curves of primary and secondary endpoints after propensity matching. **A.** Kaplan–Meier survival curves of all-cause mortality in patients with heart failure after propensity score matching. Survival curves are presented for the IMP group (blue line) and the Non-IMP group (red line). **B.** Kaplan–Meier survival curves of composite endpoint (all-cause mortality or hospitalization for heart failure) in patients with heart failure after propensity score matching. Survival curves are presented for the IMP group (blue line) and the Non-IMP group (red line).

The median follow-up was 712 days (IQR, 392–768 days), and the overall mortality was 26.35%. The median length of hospital stay was similar between the two groups (IMP group: 19 days [IQR 15–29] vs. Non-IMP group: 21 days [IQR 15–32]; P = 0.868). Kaplan–Meier survival curves of survival are shown in Figure 1. All-cause mortality in the IMP group was significantly lower than in the Non-IMP group (11.90% vs. 30.12%, log-rank P < 0.0001) (Figure 1A). The composite endpoints of all-cause mortality and hospitalization for HF were also significantly lower in the IMP group than in the Non-IMP group (29.76% vs. 47.98%; log-rank P < 0.0001) (Figure 1B). Table 2 presents the patient characteristics after propensity score matching. After propensity score matching, patients in the IMP group consistently had lower all-cause mortality and a lower incidence of the composite endpoints than those in the Non-IMP group, as shown in Figure 2A and 2B (13.74% vs. 28.24%; log-rank P = 0.0002; and 29.77% vs. 41.98%; log-rank P = 0.041, respectively).

**Table 2:** Patient characteristics after propensity score matching.


	NON-IMP	IMP	P VALUE	SMD
	
	n = 131	n = 131		

Age, years	71.40 ± 13.89	71.34 ± 14.02	0.968	0.004

Female, n (%)	74 (56.49)	77 (58.78)	0.708	0.046

History of hospitalization of HF, n (%)	50 (38.17)	50 (38.17)	>0.99	0

Hypertension, n (%)	82 (62.60)	84 (64.12)	0.798	0.032

Diabetes mellitus, n (%)	48 (36.64)	45 (34.35)	0.699	0.048

Dyslipidemia, n (%)	40 (30.53)	46 (35.11)	0.43	0.098

Hyperuricemia, n (%)	37 (28.24)	48 (36.64)	0.146	0.180

Chronic kidney disease, n (%)	58 (44.27)	55 (41.98)	0.708	0.046

Anemia, n (%)	38 (29.01)	39 (29.77)	0.892	0.017

Ischemic heart disease, n (%)	45 (34.35)	30 (22.90)	0.040	0.255

Atrial fibrillation, n (%)	54 (41.22)	66 (50.38)	0.137	0.185

BMI, kg/m^2^	23.33 ± 5.91	23.85 ± 4.80	0.061	0.097

Systolic blood pressure, mm Hg	133.36 ± 30.56	134.21 ± 33.88	0.830	0.026

Diastolic blood pressure, mm Hg	80.82 ± 21.88	81.28 ± 20.04	0.858	0.022

Heart rate, bpm	92.22 ± 25.27	90.47 ± 20.05	0.536	0.077

NYHA3/4	123 (93.89)	119 (90.83)	0.385	0.098

Dyspnea, n (%)	116 (88.55)	116 (88.55)	>0.99	0

S3, n (%)	32 (24.43)	33 (25.19)	0.886	0.018

Rales, n (%)	62 (47.33)	51 (38.93)	0.170	0.170

Edema, n (%)	93 (70.99)	104 (79.39)	0.115	0.195

Anorexia, n (%)	29 (22.14)	31 (23.66)	0.769	0.036

medication on admission				

ACEI, n (%)	21 (16.03)	30 (22.90)	0.159	0.174

ARB, n (%)	27 (20.61)	30 (22.90)	0.653	0.056

Beta blockers, n (%)	57 (43.51)	63 (48.09)	0.457	0.092

MRA, n (%)	39 (29.77)	42 (32.06)	0.688	0.050

Loop diuretics, n (%)	73 (55.73)	80 (61.07)	0.380	0.109

Thiazides, n (%)	3 (1.53)	3 (2.29)	0.651	0.056

Tolvaptan, n (%)	26 (19.85)	23 (17.56)	0.635	0.059

Nitrates, n (%)	8 (6.11)	5 (3.82)	0.391	0.106

Calcium channel blockers, n (%)	34 (25.95)	45 (34.35)	0.138	0.184

Inotropes, n (%)	11 (8.40)	13 (9.92)	0.668	0.053

SGLT2I, n (%)	7 (5.34)	7 (5.34)	>0.99	0

DOAC, n (%)	23 (17.56)	37 (28.24)	0.039	0.256

Antiplatelet, n (%)	32 (24.43)	25 (19.08)	0.294	0.130

ARNI, n (%)	0 (0.0)	0 (0.0)		

Statin, n (%)	33 (25.19)	36 (27.48)	0.674	0.052

Laboratory data				

White blood cell, /μL	7857.18 ± 3678.28	7059.06 ± 3005.53	0.056	0.238

Lymphocyte, /μL	1049.05 ± 917.28	871.18 ± 504.83	0.053	0.240

Hemoglobin, g/dL	11.62 ± 2.49	11.48 ± 2.25	0.633	0.059

Hematocrit, %	35.65 ± 7.23	35.16 ± 6.57	0.562	0.071

Blood urea nitrogen, mg/dL	29.18 ± 18.34	26.72 ± 15.81	0.246	0.144

Creatinine, mg/dL	1.523 ± 1.413	1.336 ± 0.944	0.208	0.156

eGFR, mL/min/173 m^2^	47.76 ± 24.77	49.17 ± 21.69	0.623	0.061

Uric acid, mg/dL	7.113 ± 2.587	7.141 ± 2.792	0.934	0.010

Sodium, mEq/L	137.58 ± 6.16	138.55 ± 5.73	0.189	0.163

Potassium, mEq/L	4.23 ± 0.715	4.079 ± 0.631	0.080	0.224

Chloride, mEq/L	101.69 ± 6.228	102.96 ± 5.354	0.077	0.219

Total protein, g/dL	6.266 ± 0.786	6.270 ± 0.671	0.968	0.006

Albumin, g/dL	3.06 ± 0.44	3.24 ± 0.40	0.0007	0.428

Total bilirubin, mg/dL	1.132 ± 0.777	1.315 ± 1.179	0.140	0.183

Total cholesterol, mg/dL	142.96 ± 39.43	146.14 ± 31.16	0.481	0.090

Triglycerides, mg/dL	77.72 ± 38.34	79.50 ± 35.75	0.701	0.048

HDL-cholesterol, mg/dL	44.60 ± 15.87	47.24 ± 14.55	0.167	0.173

LDL-cholesterol, mg/dL	81.93 ± 31.59	81.46 ± 28.10	0.901	0.016

BNP, pg/mL	1176.74 ± 885.93	1085.41 ± 866.55	0.488	0.104

NT-proBNP, pg/mL	14663.2 ± 23288.03	6988.56 ± 5874.83	0.018	0.452

CONUT score on admission	6.37 ± 1.546	5.90 ± 1.246	0.0068	0.334

CONUT score at discharge	6.847 ± 1.605	3.550 ± 0.499	<0.0001	2.774

Echocardiography				

LV end-diastolic diameter, mm	52.20 ± 10.35	52.36 ± 9.98	0.899	0.016

LV systolic diameter, mm	40.47 ± 12.36	40.73 ± 12.43	0.866	0.021

LV ejection fraction, %	44.21 ± 16.52	43.93 ± 16.86	0.894	0.017

LA diameter, mm	44.95 ± 9.26	45.35 ± 10.84	0.745	0.039

LA volume, mL	89.80 ± 52.09	104.75 ± 73.61	0.119	0.235

IVST, mm	9.99 ± 2.31	9.58 ± 2.09	0.132	0.186

PWT, mm	9.75 ± 1.97	9.82 ± 1.85	0.747	0.037

E wave, cm/s	86.18 ± 36.88	89.80 ± 36.79	0.439	0.098

A wave, cm/s	67.65 ± 30.10	66.88 ± 28.04	0.870	0.027

TAPSE	15.95 ± 4.51	15.68 ± 4.37	0.710	0.069

TRPG, mm Hg	28.40 ± 11.19	28.21 ± 13.38	0.902	0.015

medication at discharge				

ACEI, n (%)	54 (41.22)	61 (46.56)	0.383	0.108

ARB, n (%)	33 (25.19)	37 (28.24)	0.576	0.069

Beta blockers, n (%)	102 (77.86)	105 (80.15)	0.649	0.056

MRA, n (%)	82 (62.60)	87 (66.41)	0.519	0.080

Loop diuretics, n (%)	106 (80.92)	113 (86.26)	0.242	0.144

Thiazides, n (%)	4 (3.05)	7 (5.34)	0.353	0.114

Tolvaptan, n (%)	48 (36.64)	45 (34.35)	0.699	0.048

Nitrates, n (%)	8 (6.11)	5 (3.82)	0.391	0.106

Calcium channel blockers, n (%)	33 (25.19)	49 (37.40)	0.033	0.266

Inotropes, n (%)	20 (15.27)	18 (13.74)	0.726	0.043

SGLT2I, n (%)	20 (15.27)	22 (16.79)	0.736	0.041

DOAC, n (%)	37 (28.24)	54 (41.22)	0.027	0.275

Antiplatelet, n (%)	46 (35.11)	35 (26.72)	0.141	0.182

ARNI, n (%)	0 (0.0)	2 (2.41)	0.104	0.222

Statin, n (%)	45 (34.35)	42 (32.06)	0.694	0.049


Data are presented as mean ± SD or n (%). **Abbreviations:** ACEI, angiotensin-converting enzyme inhibitors; ARB, angiotensin II receptor blockers; ARNI, angiotensin receptor-neprilysin inhibitors; BNP, B-type natriuretic peptide; CONUT, Controlling Nutritional Status; DOAC, direct oral anticoagulants; GFR, glomerular filtration rate; HF, heart failure; IVST, interventricular septal thickness; LA, left atrial; LV, left ventricular; MRA, mineralocorticoid receptor antagonists; NYHA, New York Heart Association; PWT, posterior wall thickness; SGLT2I, sodium-glucose cotransporter 2 inhibitors; TAPSE, tricuspid annular plane systolic excursion; TRPG, tricuspid regurgitation pressure gradient.

### Predictors of nutritional improvement

LASSO logistic regression identified BMI and use of loop diuretics at admission as predictors of improvement in malnutrition. In the multivariable logistic regression model, BMI (odds ratio [OR], 1.309; 95% CI, 1.066–1.850; P = 0.043) and absence of loop diuretic use (OR, 0.087; 95% CI, 0.009–0.549; P = 0.017) were associated with improvement in malnutrition (Table 3).

**Table 3:** Multivariate logistic regression analysis for predictors of improvement in CONUT score.


	ODDS RATIO	95% CI		P VALUE

Age, per 1-year increase	1.036	0.957	1.151	0.429

BMI, per 1-kg/m^2^	1.309	1.066	1.850	0.043

Female	0.453	0.033	4.082	0.500

Loop diuretic at admission	0.087	0.009	0.549	0.017


**Abbreviations:** BMI, body mass index.

### Sensitivity and subgroup analyses

For sensitivity analysis, a multivariate Cox proportional hazards model with overlap weighting was performed using a simplified set of covariates, including age, sex, IMP, and a combined log-transformed natriuretic peptide level. This model demonstrated that CONUT score improvement was associated with lower all-cause mortality (HR, 0.357; 95% CI, 0.205–0.624; P = 0.0003) and lower rate of secondary outcome (HR, 0.669; 95% CI, 0.482–0.929; P = 0.016) (Table 4). The subgroup analyses showed the consistency of the protective effect of CONUT score improvement on all-cause mortality across clinically relevant subgroups (Figure S1). In additional sensitivity analyses using a multivariable Cox proportional hazards model, each 1-point decrease in CONUT score was associated with a lower mortality (adjusted HR 0.899, 95% CI 0.829–0.975; P = 0.01).

**Table 4:** Multivariate Cox proportional hazards model for all-cause mortality with overlap weighting.


VARIABLES	HAZARD RATIO	95% CI		P VALUE

Age, per 1-year increase	1.034	1.016	1.053	0.0002

Improvement in the CONUT score	0.357	0.205	0.624	0.0003

Male	1.470	0.969	2.229	0.070

Log natriuretic peptide, per 1 log unit increase	1.100	0.958	1.264	0.178


**Abbreviations:** CONUT, Controlling Nutritional Status.

## Discussion

In this study, among the 812 patients with HF and moderate or severe malnutrition, 168 (20.7%) showed an improvement in nutritional status by the time of discharge (i.e. their CONUT score improved to indicate no or mild malnutrition). This in-hospital improvement in CONUT score was associated with significantly better post-discharge outcomes, specifically lower all-cause mortality (primary endpoint), and a lower incidence of the composite endpoint (all-cause mortality or first HF hospitalization), compared with patients whose nutritional status did not improve. The protective association of improved CONUT score with all-cause mortality was consistent across sensitivity and subgroup analyses, underscoring the robustness of our findings. These results suggest that positive changes in nutritional score during acute HF care may translate into better prognosis. The predictors of this improvement at admission were absence of loop diuretics at admission and higher BMI at admission in LASSO logistic regression analysis. These factors likely reflect greater HF severity at baseline, which can facilitate nutritional recovery during hospitalization.

The prevalence of malnutrition assessed using the CONUT score in patients with HF has been reported to range from approximately 20% to 50%, with cutoff values varying across studies (16–17). When using a cutoff of ≥5, as the present study, previous reports have noted a malnutrition prevalence of approximately 25% (18), which is consistent with the 22.2% (812/3651) observed in our cohort. Notably, this is the first study to report the rate of in-hospital improvement in nutritional status based on a CONUT score ≥5 in hospitalized patients with HF. Existing evidence is limited to single-center studies using lower cutoff values ≥2 (19).

Our findings therefore extend the existing knowledge by showing that a substantial subset of malnourished HF patients can experience meaningful nutritional improvement during hospitalization, and by linking this improvement to better clinical outcomes. In the present study, substantial improvement in the CONUT score was observed in 20.7% of patients. This improvement likely reflects a multifactorial recovery process rather than a purely nutritional effect, encompassing the resolution of congestion and inflammation along with possible nutritional replenishment.

The mechanisms underlying nutritional score improvement during HF hospitalization are likely multifactorial. Effective HF treatment can relieve congestion and systemic inflammation, which in turn may restore appetite and gastrointestinal function (20,21). Moreover, sustained stimulation of the sympathetic nerve system and the renin-angiotensin-system promotes protein catabolism through pro-inflammatory cytokine release, oxidative stress, and mitochondrial dysfunction (22). Angiotensin II has been implicated in skeletal muscle wasting through upregulation of catabolic pathways, including the ubiquitin-proteasome system and suppression of anabolic signaling (23). By initiating or up-titrating beta-blockers and renin-angiotensin system inhibitors, inpatient HF care may blunt these neurohormonal pathways, potentially mitigating muscle wasting and HF-related cachexia. The nutritional score improvement observed in our study may reflect this combined recovery process.

Despite the usefulness of the CONUT score for risk stratification, it is important to acknowledge that this was originally developed for the non-HF populations and may be influenced by HF-related factors. Each component of the CONUT score can be altered by pathophysiology of HF, such as hemodilution, liver dysfunction, and chronic inflammation (24). Therefore, the CONUT score in patients with HF may reflect disease severity rather than purely nutritional deficits. This means that improving CONUT score during treatment could parallel improvements in the underlying HF condition. We attempted to account for disease severity by adjustment techniques, but residual confounding may still exist. Nonetheless, whether improvement in CONUT score represents true nutritional replenishment or simply an epiphenomenon of HF recovery, it remains a useful integrated marker of patient status that correlates with outcomes.

In an exploratory analysis of predictors of improvement in the CONUT score, a higher BMI was associated with a greater likelihood of improvement in the CONUT score, whereas use of loop diuretics at admission were less likely to show improvement. These findings align with previous observations that weight loss and aggressive diuretic use in HF are markers of advanced disease and elevated inflammatory state (25,26). Chronic inflammation is a key feature of HF that can drive malnutrition through increased catabolism and anorexia (27). Patients with milder disease or preserved BMI may respond better to standard inpatient care, with improvements in their nutritional indices. Overall, our results support the idea that longitudinal assessment of nutritional status adds prognostic information in HF. Not only does a single malnutrition score at admission carry risk stratification value, but the trajectory of nutritional status (improving or not) appears to further stratify patients’ risk of adverse outcomes.

Hersberger et al. reported that individualized nutritional interventions reduced mortality in patients with HF and malnutrition, as assessed using the Nutritional Risk Screening (28). While this randomized trial suggests that nutritional intervention may be beneficial in selected patients, our observational findings should be interpreted cautiously. Because no specialized nutritional program was mandated in our study, the observed improvements in CONUT score likely reflect overall clinical recovery during hospitalization, including optimized medical management and rehabilitation, rather than a direct effect of targeted nutritional therapy. The mechanisms underlying CONUT score improvement during hospitalization may differ between patients with de novo acute HF and those with acute decompensation of chronic HF, with the former more likely reflecting resolution of acute illness rather than reversal of chronic catabolic states. Importantly, our results should be considered hypothesis-generating with respect to nutritional interventions. Patients whose CONUT score does not improve during hospitalization may represent a high-risk subgroup in whom persistent vulnerability is captured by nutritional indices. Whether targeted nutritional strategies after discharge can improve outcomes beyond standard HF care warrants investigation in well-designed prospective trails.

### Limitations

The present study has several limitations. First, we lacked detailed data on patients’ dietary intake, we could not quantify how caloric or protein consumption correlated with CONUT score changes. Second, including BNP and NT-proBNP in propensity score matching would have resulted in a substantial reduction in sample size; therefore, these variables were not incorporated in the matching procedure. However, the multivariable Cox hazard model with overlap weighting included log-transformed natriuretic peptide as a sensitive analysis and yielded results consistent with those of the propensity score matching analysis. Nevertheless, residual confounding related to heart failure severity, including congestion status and in-hospital treatment intensity, cannot be fully excluded and should be considered when interpreting the findings. Third, as this study was a sub-analysis of a Japanese cohort, the findings may not be generalizable to other populations or healthcare systems, where patient characteristics, nutritional background, and patterns of acute HF management, including length of hospital stay, may differ substantially. Fourth, the JROADHF-NEXT registry includes only patients who survived through hospital discharge; therefore, survivorship bias is inherent to the study design. Patients who did not survive through discharge likely had more severe HF and poorer nutritional status. As a result, the observed association may underestimate the true prognostic impact of malnutrition. Fifth, we clarified that LASSO selection reflects predictive importance rather than linearity and acknowledged the possibility of non-linear effects of BMI as a limitation. Sixth, we were unable to distinguish between de novo acute HF and acute decompensation of chronic HF because this information was not available in the registry. Differences in nutritional reserve and disease burden between these entities may influence CONUT score trajectories and should be considered when interpreting the findings. Finally, as an observational study, our findings show associations but cannot prove causation; unmeasured confounder may partly explain the link between nutritional improvement and outcomes.

## Conclusion

In hospitalized patients with acute HF and moderate to severe malnutrition, improvement in CONUT score during hospitalization was associated with lower post-discharge mortality and rehospitalization. These findings suggest that dynamic changes in nutritional indices may function as integrated markers of clinical recovery and prognosis, although causal effects of nutritional interventions cannot be inferred from this observational association.

## Data Accessibility Statement

The original contributions presented in the study are included in the article, and further inquiries can be directed to the corresponding author.

## Additional File

The additional file for this article can be found as follows:

10.5334/gh.1534.s1 Figure S1. Forest plot of subgroup analysis for all-cause mortality according to CONUT improvement (overlap weighting).
