# Supplementary material for: Prognostic Value of In-Hospital Nutritional Status Improvement in Heart Failure: Insights From JROADHF-NEXT Registry
Source: Glob Heart. 2026 Mar 13;21(1):20. doi: 10.5334/gh.1534 (PMC12985903; doi:10.5334/gh.1534)

Figure S1: Forest plot of subgroup analysis for all-cause mortality according to CONUT improvement (overlap weighting).

HRs or CI values extending beyond the axis limits are truncated. HRs were estimated with overlap weighting; results were consistent across subgroups with no significant interactions.

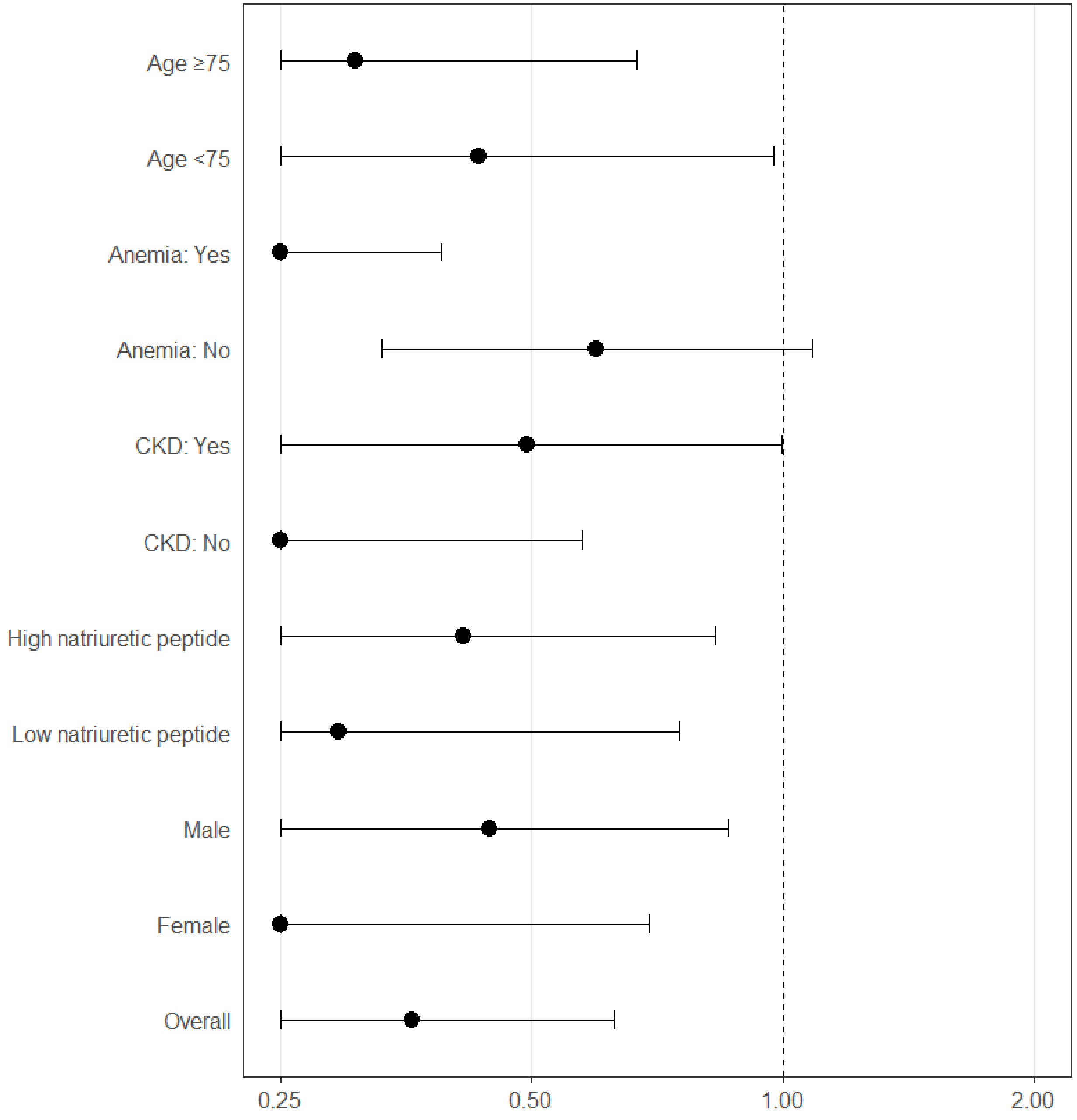

Supplement: Figure S1. — Forest plot of subgroup analysis for all-cause mortality according to CONUT improvement (overlap weighting). [file gh-21-1-1534-s1.pdf]
